# Supplementary material for: Intolerance upon statin rechallenge: A systematic review and meta-analysis of randomized controlled trials
Source: PLoS One. 2023 Dec 21;18(12):e0295857. doi: 10.1371/journal.pone.0295857 (PMC10735036; doi:10.1371/journal.pone.0295857)
Supplement: S1 Text — (PDF) [file pone.0295857.s001.pdf]

Intolerance upon statin rechallenge: a systematic review and meta-analysis of randomized controlled trials
S1 Text: Search strategy

Medline, Embase, CINAHL Plus, Scopus, Cochrane CENTRAL trial registry

Ovid MEDLINE(R) ALL <1946 to April 27, 2023>
Date searched: May 1, 2023

1 hydroxymethylglutaryl-coa reductase inhibitors/ or atorvastatin/ or lovastatin/ or meglutol/ or pravastatin/ or rosuvastatin calcium/ or simvastatin/ 46370
2 (atorvastatin or simvastatin or rosuvastatin or fluvastatin or pravastatin or pitavastatin or lovastatin or cerivastatin or statin or statins or hydroxymethylglutaryl-CoA reductase inhibitor*).mp. 70578
3 1 or 2 70713
4 nocebo.mp. 1152
5 ((restart* or re-start* or rechalleng* or re-challeng* or resum*) adj10 (atorvastatin or simvastatin or rosuvastatin or fluvastatin or pravastatin or pitavastatin or lovastatin or cerivastatin or statin or statins or hydroxymethylglutaryl-CoA reductase inhibitor*)).mp. 156
6 ((atorvastatin or simvastatin or rosuvastatin or fluvastatin or pravastatin or pitavastatin or lovastatin or cerivastatin or statin or statins or hydroxymethylglutaryl-CoA reductase inhibitor*) and (intoleran* or myopath* or myalgi* or ((muscle or muscular) and (side effect* or adverse or issues or problems or reaction or reactions or symptoms)))).ti,bt,kf.1214
7 or/4-6 2432
8 3 and 7 1332
9 exp Clinical trial/ or (randomi* or randomly or (random adj4 (allocat* or distribut* or assign*)) or placebo or trial or groups or subgroups or (phase adj1 ("3" or "2" or "1" or III or II or I))).tw. or dt.fs. 6002128
10 8 and 9 624

Embase (Ovid Interface) <1974 to 2023 April 28>
Date searched: May 1, 2023

1 exp hydroxymethylglutaryl coenzyme A reductase inhibitor/ 189030
2 (atorvastatin or simvastatin or rosuvastatin or fluvastatin or pravastatin or pitavastatin or lovastatin or cerivastatin or statin or statins or hydroxymethylglutaryl-CoA reductase inhibitor*).mp. 144754
3 1 or 2 203323
4 nocebo.mp. 1741
5 ((restart* or re-start* or rechalleng* or re-challeng* or resum*) adj10 (atorvastatin or simvastatin or rosuvastatin or fluvastatin or pravastatin or pitavastatin or lovastatin or cerivastatin or statin or statins or hydroxymethylglutaryl-CoA reductase inhibitor*)).mp. 293

6 ((atorvastatin or simvastatin or rosuvastatin or fluvastatin or pravastatin or pitavastatin or lovastatin or cerivastatin or statin or statins or hydroxymethylglutaryl-CoA reductase inhibitor*) and (intoleran* or myopath* or myalgi* or ((muscle or muscular) and (side effect* or adverse or issues or problems or reaction or reactions or symptoms)))).ti,bt,kf.1974
7 4 or 5 or 6 3845
8 3 and 7 2216
9 exp Clinical trial/ or (randomi* or randomly or (random adj4 (allocat* or distribut* or assign*)) or placebo or trial or groups or subgroups or (phase adj1 ("3" or "2" or "1" or III or II or I))).tw. 6045893
10 8 and 9 635

CINAHL Plus with Full Text (EBSCOhost interface)
Date searched: May 1, 2023
Results: 108 results

S1 (MH "Statins+") or atorvastatin or simvastatin or rosuvastatin or fluvastatin or pravastatin or pitavastatin or lovastatin or cerivastatin or statin* or hydroxymethylglutaryl-CoA reductase inhibitor* (25,453)
S2 (nocebo or ((restart* or re-start* or rechalleng* or re-challeng* or resum*) N10 (atorvastatin or simvastatin or rosuvastatin or fluvastatin or pravastatin or pitavastatin or lovastatin or cerivastatin or statin or statins or hydroxymethylglutaryl-CoA reductase inhibitor*))) OR (TI((atorvastatin or simvastatin or rosuvastatin or fluvastatin or pravastatin or pitavastatin or lovastatin or cerivastatin or statin or statins or hydroxymethylglutaryl-CoA reductase inhibitor*) and (intoleran* or myopath* or myalgi* or ((muscle or muscular) and (side effect* or adverse or issues or problems or reaction or reactions symptoms))))) (1000 results)
S3 ((MH "Clinical Trials+") OR (MH "Community Trials") or randomi* or "randomly" or ("random" N4 (allocat* or distribut* or assign*)) or "placebo" or "trial" or "groups" or "subgroups" OR or (phase N1 ("3" or "2" or "1" or III or II or I)) or quasirandom* OR TI(RCT)) (1,099,375)
S4 S1 AND S2 AND S3 108 results

Scopus (Advanced search)
Date searched: May 1, 2023
Results: 405

TITLE-ABS-KEY(atorvastatin or simvastatin or rosuvastatin or fluvastatin or pravastatin or pitavastatin or lovastatin or cerivastatin or statin or statins or hydroxymethylglutaryl-CoA-reductase-inhibitor*) AND (TITLE-ABS-KEY(nocebo or ((restart* or re-start* or rechalleng* or re-challeng* or resum*) W/10 (atorvastatin or simvastatin or rosuvastatin or fluvastatin or pravastatin or pitavastatin or lovastatin or cerivastatin or statin or statins or hydroxymethylglutaryl-CoA-reductase-inhibitor*))) OR TITLE((atorvastatin or simvastatin or rosuvastatin or fluvastatin or pravastatin or pitavastatin or lovastatin or cerivastatin or statin or statins or hydroxymethylglutaryl-CoA-reductase-inhibitor*) and (intoleran* or myopath* or

myalgi* or ((muscle or muscular) and (side-effect* or adverse or issues or problems or reaction or reactions or symptoms))))) AND (TITLE-ABS-KEY ({Clinical-trial} OR {controlled-trial} OR randomi* OR {randomly} OR (random W/4 (allocat* OR distribut* OR assign*)) OR {placebo} OR {trial} OR {groups} OR {subgroups} or (phase W/1 ("3" or "2" or "1" or III or II or I))) OR TITLE (rct))

Cochrane CENTRAL Trial Registry (Trials database only)
Date searched: May 1, 2023
Results: 199

#1: (atorvastatin or simvastatin or rosuvastatin or fluvastatin or pravastatin or pitavastatin or lovastatin or cerivastatin or statin or statins or hydroxymethylglutaryl-CoA-reductase-inhibitor):ti,ab,kw
#2: (nocebo or ((restart* or re-start* or rechalleng* or re-challeng* or resum*) NEAR/10 (atorvastatin or simvastatin or rosuvastatin or fluvastatin or pravastatin or pitavastatin or lovastatin or cerivastatin or statin or statins or hydroxymethylglutaryl-CoA-reductase-inhibitor))):ti,ab,kw
#3 ((atorvastatin or simvastatin or rosuvastatin or fluvastatin or pravastatin or pitavastatin or lovastatin or cerivastatin or statin or statins or hydroxymethylglutaryl-CoA-reductase-inhibitor) and (intoleran* or myopath* or myalgi* or ((muscle or muscular) and (side-effect or adverse or issues or problems or reaction or reactions or symptoms)))):ti
#4 #1 and (#2 OR #3)
