# Supplementary material for: Intolerance upon statin rechallenge: A systematic review and meta-analysis of randomized controlled trials
Source: PLoS One. 2023 Dec 21;18(12):e0295857. doi: 10.1371/journal.pone.0295857 (PMC10735036; doi:10.1371/journal.pone.0295857)
Supplement: S1 Fig — (PDF) [file pone.0295857.s004.pdf]

Intolerance upon statin rechallenge: a systematic review and meta-analysis of randomized controlled trials

S1 Figure: Subgroup analysis - exposure

	Experimental		Control			Risk ratio
**Study or Subgroup**	**Events**	**Total**	**Events**	**Total**	**Weight**	**M-H, Fixed, 95% CI**
**1.2.1 Lesser exposure**						
Howard 2021	24	60	20	60	8.6%	1.20 [0.75 , 1.93]
Joy 2014	1	8	1	8	0.4%	1.00 [0.07 , 13.37]
Kennedy 2011	3	17	2	17	0.9%	1.50 [0.29 , 7.87]
Kristiansen 2021	20	77	12	77	5.2%	1.67 [0.88 , 3.17]
**Subtotal (95% CI)**		**162**		**162**	**15.1%**	**1.37 [0.95 , 1.99]**
Total events:	48		35			
Heterogeneity: Chi^2 = 0.73, df = 3 (P = 0.87); I^2 = 0%						
Test for overall effect: Z = 1.67 (P = 0.09)						
**1.2.2 Greater exposure**						
Herrett 2021	18	200	15	200	6.5%	1.20 [0.62 , 2.31]
Nissen 2016	257	480	178	483	76.4%	1.45 [1.26 , 1.68]
Stein 2008	2	64	5	66	2.1%	0.41 [0.08 , 2.05]
**Subtotal (95% CI)**		**744**		**749**	**84.9%**	**1.41 [1.22 , 1.62]**
Total events:	277		198			
Heterogeneity: Chi^2 = 2.66, df = 2 (P = 0.26); I^2 = 25%						
Test for overall effect: Z = 4.77 (P < 0.00001)						
**Total (95% CI)**		**906**		**911**	**100.0%**	**1.40 [1.23 , 1.60]**
Total events:	325		233			
Heterogeneity: Chi^2 = 3.45, df = 6 (P = 0.75); I^2 = 0%						
Test for overall effect: Z = 5.03 (P < 0.00001)						
Test for subgroup differences: Chi^2 = 0.02, df = 1 (P = 0.90), I^2 = 0%						

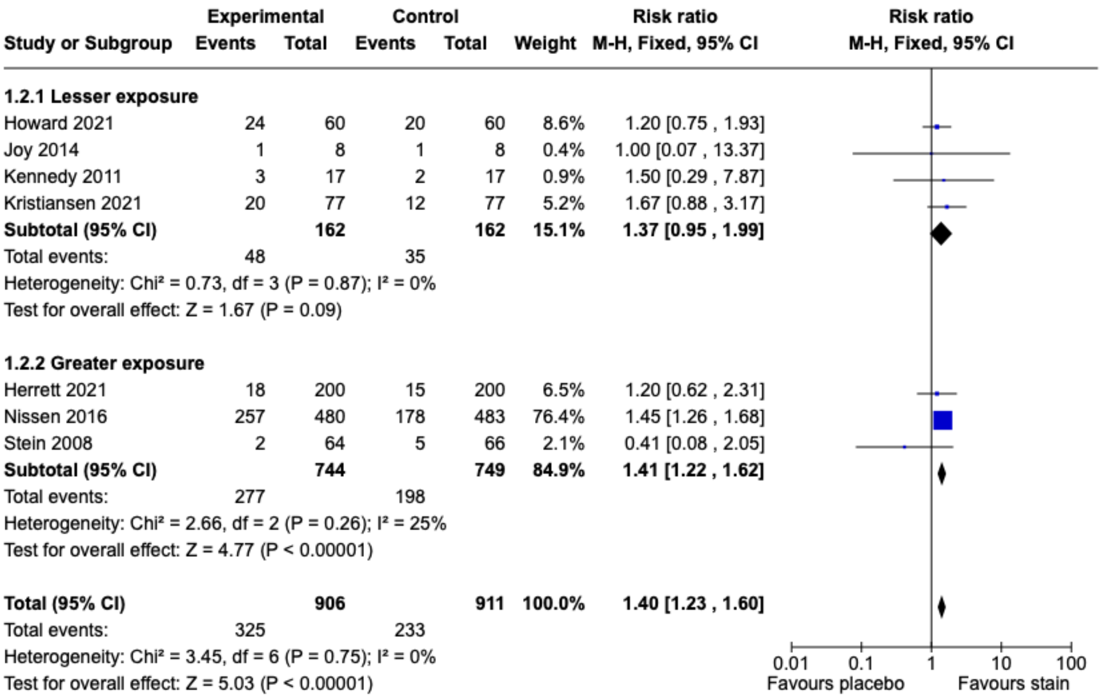

Prepared August 2023
