# Supplementary material for: Intolerance upon statin rechallenge: A systematic review and meta-analysis of randomized controlled trials
Source: PLoS One. 2023 Dec 21;18(12):e0295857. doi: 10.1371/journal.pone.0295857 (PMC10735036; doi:10.1371/journal.pone.0295857)
Supplement: S2 Fig — (PDF) [file pone.0295857.s005.pdf]

Intolerance upon statin rechallenge: a systematic review and meta-analysis of randomized controlled trials

S2 Figure: Subgroup analysis - intolerance definition

	Experimental		Control			Risk ratio
**Study or Subgroup**	**Events**	**Total**	**Events**	**Total**	**Weight**	**M-H, Fixed, 95% CI**
**1.3.1 Stops and withdrawals**						
Herrett 2021	18	200	15	200	6.5%	1.20 [0.62 , 2.31]
Howard 2021	24	60	20	60	8.6%	1.20 [0.75 , 1.93]
Joy 2014	1	8	1	8	0.4%	1.00 [0.07 , 13.37]
Kennedy 2011	3	17	2	17	0.9%	1.50 [0.29 , 7.87]
Stein 2008	2	64	5	66	2.1%	0.41 [0.08 , 2.05]
**Subtotal (95% CI)**		**349**		**351**	**18.5%**	**1.12 [0.78 , 1.61]**
Total events:	48		43			
Heterogeneity: Chi² = 1.74, df = 4 (P = 0.78); I² = 0%						
Test for overall effect: Z = 0.61 (P = 0.54)						
**1.3.2 Myalgia symptoms**						
Kristiansen 2021	20	77	12	77	5.2%	1.67 [0.88 , 3.17]
Nissen 2016	257	480	178	483	76.4%	1.45 [1.26 , 1.68]
**Subtotal (95% CI)**		**557**		**560**	**81.5%**	**1.47 [1.27 , 1.69]**
Total events:	277		190			
Heterogeneity: Chi² = 0.17, df = 1 (P = 0.68); I² = 0%						
Test for overall effect: Z = 5.34 (P < 0.00001)						
**Total (95% CI)**		**906**		**911**	**100.0%**	**1.40 [1.23 , 1.60]**
Total events:	325		233			
Heterogeneity: Chi² = 3.45, df = 6 (P = 0.75); I² = 0%						
Test for overall effect: Z = 5.03 (P < 0.00001)						
Test for subgroup differences: Chi² = 1.85, df = 1 (P = 0.17), I² = 45.8%						

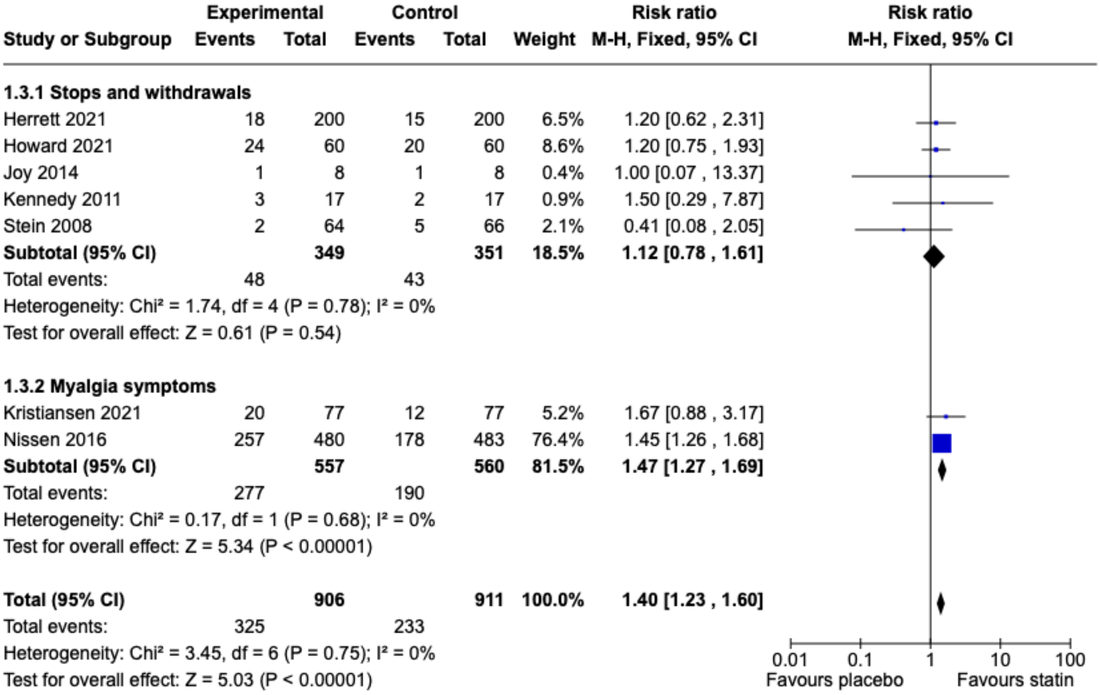

Prepared August 2023
