# Supplementary material for: Intolerance upon statin rechallenge: A systematic review and meta-analysis of randomized controlled trials
Source: PLoS One. 2023 Dec 21;18(12):e0295857. doi: 10.1371/journal.pone.0295857 (PMC10735036; doi:10.1371/journal.pone.0295857)
Supplement: Attachment — Submitted filename: 23Nov07 rebuttal letter.pdf [file pone.0295857.s006.pdf]

UNIVERSITY OF ALBERTA

Nov 7, 2023

Vikramaditya Samala Venkata
Academic Editor
PLOS ONE

Dear Dr. Venkata:

Please find below our response to the reviewer feedback in bold. If you require any additional clarification/revisions, please let us know.

1. Authors have selected a very important topic to study. But results need to be explained better. In the results section: we need to report the results with a link to the figure and then talk about them in the discussion.
   Directly in the discussion, authors reported that
   "Only 35.9% of participants were intolerant of statins on rechallenge compared to 25.6% who were intolerant on placebo"
   "Mean myalgia/global symptom score was higher on statins, but only marginally so, and with an upper bound on the 95% confidence interval (3.67 on a 100-point scale)"
   Where are these results documented, in which figure. Results need to be reported clearly in the results section with a link to the figure and then we can discuss these results in discussion section.

   Thank you for bringing this to our attention. These results are in Figure 2 (meta-analysis of intolerance of statin versus placebo) and Figure 4 (meta-analysis of mean myalgia/global symptom score on statin versus placebo). We have reorganized the results section to make it easier to follow with the figures directly below the discussion.

2. Average dose of statin and symptom's upon rechallenge is not clear from the study

   We decided to provide statin intensity rather than dose in the results section (paragraph 2 and table 1) to make it easier to compare the statin dose between trials. The dose is provided in S2 table: Full data extraction.

   We describe the symptoms measured and the scale used in the results section (paragraph 3 and table 1). The meta-analysis provides the mean and standard deviation of the scores for each study for both placebo and statin (on a 100-point scale).

Sincerely,

Roni Kraut

Roni Kraut CPA, MD, CCFP, MSc
Assistant Clinical Professor, University of Alberta

Department of Family Medicine
College of Health Sciences
Faculty of Medicine & Dentistry
6-10 University Terrace
University of Alberta
Edmonton AB, Canada T6G 2T4

T 780.492.8306 / 8102
F 780.492.2593
rkraut@ualberta.ca
ualberta.ca//family-medicine
